# Metastatic Renal Cell Carcinoma Presenting as Gastric Ulcer: Case Report and Literature Review

**DOI:** 10.1155/2017/2509294

**Published:** 2017-06-21

**Authors:** Alhareth Al Juboori, Satinder Kaur, Atigadda Reddy

**Affiliations:** ^1^University of Missouri School of Medicine, Columbia, MO, USA; ^2^Veterans Affairs Medical Center, University of Nevada School of Medicine, Reno, NV, USA; ^3^University of Nevada School of Medicine, Reno, NV, USA

## Abstract

Renal cell carcinoma (RCC) accounts for approximately 3% of all adult malignancies. True gastrointestinal metastases, specifically to gastric wall, have been rarely observed. Herein we describe a case of delayed metastasis to gastric wall occurring more than a decade after previously curative nephrectomy for RCC. A 67-year-old male with history of right radical nephrectomy in 2001 for RCC was found to have an asymptomatic right lower lobe solitary lung mass upon routine follow-up in 2011, with final biopsy results showing metastatic RCC for which he was treated accordingly. In 2014, patient was evaluated for dyspepsia with microcytic anemia and underwent an esophagogastroduodenoscopy and colonoscopy. EGD revealed a solitary one-centimeter atypical ulcer in the posterior mid gastric body with biopsy results being consistent with metastatic RCC. Our literature review has yielded thirty-six reported cases of RCC in association with gastric wall metastases.

## 1. Introduction

We report a case of delayed metastasis to the gastric wall occurring more than a decade after curative nephrectomy for renal cell carcinoma (RCC), which manifested as an atypical gastric ulcer. RCC accounts for about three percent of all adult malignancies and has a high propensity for local and systemic spread [1]. Commonly described metastatic sites include lung (45.2%), bone (29.5%), lymph nodes (21.8%), liver (20.3%), adrenal (8.9%), and brain (8.1%) [2]. True gastrointestinal metastases from RCC specifically to the gastric wall are extremely rare.

## 2. Case Presentation

**Figure 1:**
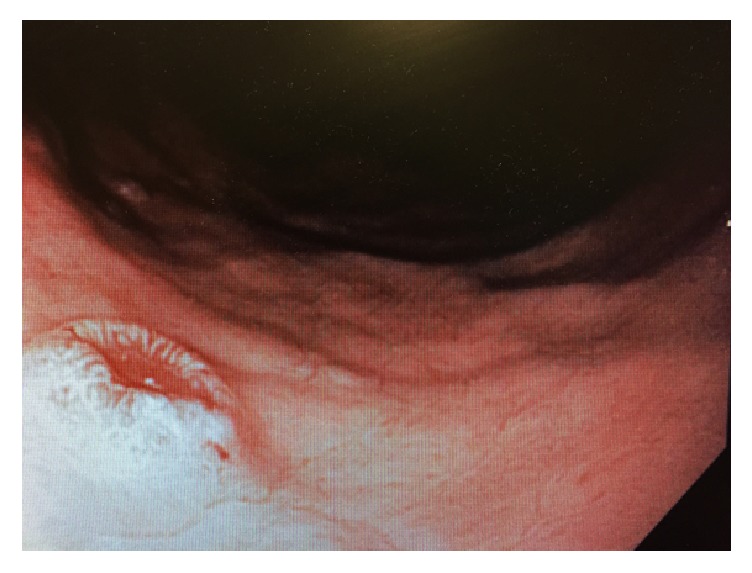
Malignant appearing gastric ulcer during endoscopic evaluation.

**Figure 2:**
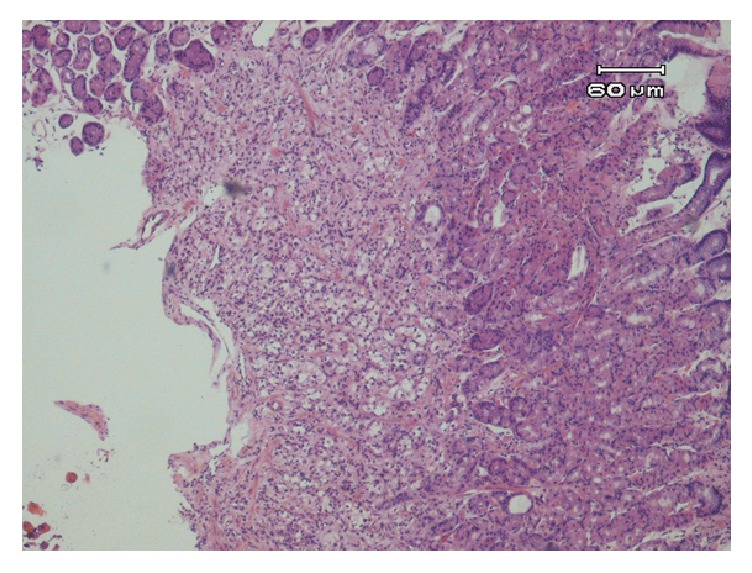
Gastric ulcer biopsy showing clear cell RCC consistent with metastatic RCC to stomach.

A 67-year-old Caucasian male with history of RCC stage I T1N0M0 grade II underwent a curative right radical nephrectomy in 2001. During routine follow-up in 2011 patient was found to have an asymptomatic right lower lobe solitary lung mass. A transbronchial biopsy of the lung lesion was performed and was consistent with metastatic RCC for which he was started on sunitinib. In April 2014, patient was evaluated for dyspepsia and microcytic anemia, for which an esophagogastroduodenoscopy (EGD) and colonoscopy were done. EGD revealed a solitary one- centimeter atypical ulcer located in the posterior mid gastric body with raised and friable margins and a clean ulcer base (Figure 1). Ulcer margin biopsies revealed metastatic clear cell renal cell carcinoma with histologic features similar to those of the primary renal tumor and lung lesion. Histopathology showed epithelial tumor cells with clear cytoplasm, consistent with clear cell RCC (Figure 2). Carbonic anhydrase XI stain was positive whereas both CD117 and CK7 stains were negative, and these are confirmatory stains for clear cell RCC. Colonoscopy revealed uncomplicated diverticulosis and no other potential causes of microcytic anemia. Decision was made to initiate Everolimus. Soon afterwards, patient was admitted for a right femur fracture requiring surgical fixation, and a radionucleotide bone scan was done and revealed multiple skeletal metastases.

During the ensuing ten months, despite Everolimus therapy patient went on to develop progressive metastatic disease involving the liver and intrapelvic structures in addition to his skeletal metastases. Due to lack of therapeutic response and disease progression patient opted for hospice care and subsequently expired in February 2015—some 14 years after the initial diagnosis of RCC.

## 3. Discussion

RCC is a primary renal malignancy, which originates in the renal cortex from proximal tubular epithelium, and is known for its aggressive metastatic nature. Gastrointestinal metastases, specifically to gastric wall from RCC, are a rare phenomenon but have been commonly described in association with primary tumors of the breast, lung, and melanoma [3, 4]. In a case report by Kim et al. thirty-six cases of RCC with gastric wall metastases were identified [5]. Clinical manifestations most commonly associated in patients with gastric metastases included anemia, gastrointestinal bleeding, dyspepsia, and epigastric pain [5, 6]. Even though our patient did not have overt gastrointestinal bleeding, his presentation of microcytic anemia without colonic etiology was felt to be related to the ulcerated gastric wall metastatic lesion seen on EGD. Their case report also highlights that metastases are most common in the gastric body and are more likely to be single rather than multiple.

With regard to the management of RCC treatment includes radial nephrectomy in cases with no metastasis, and clinical studies are experimenting with cytoreductive nephrectomy and use of antiangiogenic agents, chemotherapy, or immunotherapy in cases of metastatic disease [7]. In patients with metastatic RCC to the stomach treatment modality is still unclear. Each patient is reviewed on a case-by-case basis and evaluated for endoscopic mucosal resection, surgical resection, chemotherapy, or immunotherapy depending on stage of the disease.

In summary, we have described an unusual case of gastric wall metastasis, thirteen years after a clinically curative radical nephrectomy, manifesting as an atypical gastric ulcer with microcytic anemia. In the absence of intra-abdominal recurrent RCC this is likely to represent a true hematogenous metastasis to the gastric mucosa and subsequent ulceration. Additionally, our case illustrates the unusual tumor behavior of RCC to result in delayed metastases occurring more than a decade after removal of the primary tumor.
